# Special Issue “*Aspergillus fumigatus*: From Diagnosis to Therapy”

**DOI:** 10.3390/jof2040031

**Published:** 2016-12-08

**Authors:** William J. Steinbach

**Affiliations:** 1 Division of Pediatric Infectious Diseases, Department of Pediatrics, Duke University Medical Center, Durham, NC 27710, USA; bill.steinbach@duke.edu; Tel.: +919-681-1504; 2 Department of Molecular Genetics and Microbiology, Duke University Medical Center, Durham, NC 27710, USA

*Aspergillus fumigatus* is an enigmatic pathogen. Depending on the immune status of the infected host, this fungus can cause saprophytic, allergic, or deadly invasive disease. There are few pathogens that are the etiology for such a wide spectrum of clinical presentation. The nine articles in this unique supplement cover the breadth of *Aspergillus fumigatus*. Diagnosis remains controversial and difficult—there is no simple and fully reproducible assay that signals definitive disease, such as an easily acquired blood culture for so many other pathogens. Culture on mycological media is still accepted as a gold-standard, but the ability to obtain a sample from a sterile site in a fragile patient is increasingly more difficult and rarely possible. Cultures from non-sterile sites have uncertain clinical predictive value. Thus, diagnosis has classically been relegated to indirect findings through radiology, or in the last decade newer molecular testing methodologies. While biomarkers such as the galactomannan assay, or the more fungal-general 1,3-β-d-glucan assay, have opened newer avenues for diagnosis, most testing is very specific to the host. The biomarkers have known subpopulations where they are effective, and unfortunately groups of patients where the results are more questionable. *Aspergillus* PCR is advancing through a large consortium group, with the future goals focused on standardized and reproducible techniques and results.

The clinical presentations of disease vary by age, as outlined in the article focusing on the nuances of pediatric aspergillosis. The acute invasive form differs substantially in phenotype as well as therapeutic approach from allergic bronchopulmonary aspergillosis or chronic pulmonary aspergillosis. The strategies for allergic aspergillus rhinosinusitis are also evolving, and all Aspergillus disease is increasingly affected by the worldwide pandemic of increasing azole resistance.

The future for these diseases is promising—newer treatment approaches based on available evidence and novel diagnostic strategies. This supplement was designed to encapsulate the spectrum of the clinical disease in a variety of host situations, coupled with the latest thoughts on diagnosis and antifungal management. The authors are all leaders in those respective fungal subfields, offering the latest tools to combat *Aspergillus fumigatus*.

